# Effects of school‐based mindfulness training on emotion processing and well‐being in adolescents: evidence from event‐related potentials

**DOI:** 10.1111/desc.12646

**Published:** 2018-01-22

**Authors:** Kevanne Louise Sanger, Guillaume Thierry, Dusana Dorjee

**Affiliations:** ^1^ Centre for Mindfulness Research and Practice Bangor University Bangor Gwynedd UK; ^2^ School of Psychology Bangor University Bangor Gwynedd UK

## Abstract

In a non‐randomized controlled study, we investigated the efficacy of a school‐based mindfulness curriculum delivered by schoolteachers to older secondary school students (16–18 years). We measured changes in emotion processing indexed by P3b event‐related potential (ERP) modulations in an affective oddball task using static human faces. ERPs were recorded to happy and sad face oddballs presented in a stimulus stream of frequent faces with neutral expression, before and after 8 weeks of mindfulness training. Whilst the mean amplitude of the P3b, an ERP component typically elicited by infrequent oddballs, decreased between testing sessions in the control group, it remained unchanged in the training group. Significant increases in self‐reported well‐being and fewer doctor visits for mental health support were also reported in the training group as compared to controls. The observed habituation to emotional stimuli in controls thus contrasted with maintained sensitivity in mindfulness‐trained students. These results suggest that in‐school mindfulness training for adolescents has scope for increasing awareness of socially relevant emotional stimuli, irrespective of valence, and thus may decrease vulnerability to depression.

**RESEARCH HIGHLIGHTS**

- Mindfulness training was associated with maintained P3b mean amplitudes to facial target stimuli, indicating sustained sensitivity to socially relevant, affective stimuli.
- Trained students reported higher well‐being despite mindfulness course engagement being correlated with greater stress awareness.
- Self‐reported changes in empathy correlated significantly with changes in P3b to emotional faces across groups.

## 1 INTRODUCTION

Adolescence is a time of stress vulnerability, with high academic demands and social pressures. It is also considered a sensitive point for depression onset, with the reward system of the brain (ventral striatum) maturing before the prefrontal regions (PFC, ACC) that balance decision‐making and regulate behaviour (Ernst, Pine, & Hardin, 2006). According to the Rescorla‐Wagner learning model, the dopaminergic response to reward means that when a distal goal is not achieved, dopamine expression is suppressed, which can lead to prolonged suppression of the reward system (Schultz, 2010). In adolescents, this reward suppression combined with an immature PFC can result in a weak ability to regulate emotions, triggering depression (Weir, Zakama, & Rao, 2012). Indeed the World Health Organization lists depression as the top cause of years lost to disability in adolescents (WHO, 2015), and the National Institute for Care Excellence recommends that schools develop procedures to detect early symptoms of depression (NICE, 2015).

In an attempt to promote mental health, some schools are introducing mindfulness‐based interventions into their curricula. The practice of secular mindfulness can be described as intentional attending to the present moment experience, with a kind and accepting attitude (Kabat‐Zinn, 1994). Improvements in anxiety and recurrent depression after mindfulness training are well documented in adults (e.g., Williams et al., 2014), and psychological benefits of mindfulness training have also been shown in adolescents (Biegel, Brown, Shaprio, & Schubert, 2009; Kuyken et al., 2013). Specifically, initial studies of school‐based programmes have found improvements in perceived stress and well‐being, as well as reductions in anxiety and depression (Huppert & Johnson, 2010; Kuyken et al., 2013; Metz et al., 2013; Raes, Griffith, Van der Gucht, & Williams, 2014; see Zenner, Herrnleben‐Kurz, & Walach, 2014, for an overview of school‐based research).

Mindfulness‐based interventions have been shown to improve healthy reflection on distressing memories in adults (Hargus, Crane, Barnhofer, & Williams, 2010), and to attenuate interference from negative stimuli in university students (Eddy, Brunyé, Tower‐Richardi, Mahoney, & Taylor, 2015; Ortner, Kilner, & Zelazo, 2007). This is relevant to depression research, where clinical groups have been found to demonstrate a negativity bias in autobiographical memory, and in response to visual and auditory affective stimuli (Gotlib, & Neubauer, 2000). Mindful and depressed participants also seem to differ in how they relate to others. Adults with major depression disorder (MDD) tend to struggle with cognitive empathy, including perspective taking and theory of mind (Schreiter, Pijnenborg, & Aan Het Rot, 2013), while mindfulness training may increase empathy (Block‐Lerner, Adair, Plumb, Rhatigan, & Orsillo, 2007; Music, 2014). Consistent with this view, Hölzel et al. (2011) reported increased grey matter concentration in the temporal‐parietal junction (TPJ), a key area for social cognition and perspective taking, after mindfulness‐based stress reduction (MBSR). However, there is currently no evidence to support the existence of similar benefits in adolescents.

When considering how mindfulness may impact on neural correlates of depression or depression vulnerability in adolescence, we can assume a divergent pattern of activation between depression and mindfulness‐based change (Deng, Li, & Tang, 2014; Desrosiers, Klemanski, & Nolen‐Hoeksema, 2013; Way, Creswell, Eisenberger, & Lieberman, 2010). There are different models of depression, upon which we can hypothesize a potential impact of mindfulness training in adolescents. The Emotion Context Insensitivity (ECI) model, for example, proposes that depression is characterized by a lack of response to both positive and negative emotional stimuli, a coping mechanism preventing further reactivity in individuals experiencing chronic, high‐intensity stress (Rottenberg, Gross, & Gotlib, 2005). A meta‐analysis supporting this model (Bylsma, Morris, & Rottenberg, 2008) shows that MDD groups exhibit dampened responses to both positive and negative stimuli compared to controls. In the adolescent literature, Blom et al. (2015) reported that depressed adolescents showed significantly reduced anterior/middle insular cortex activation when viewing sad as opposed to happy faces, as compared to controls, an effect considered a developmental signature of depression (see Smith, Steinberg, & Chein, 2013).

It has been argued that mindfulness encourages openness to both positive and negative experience whilst minimizing reactivity or rumination. It can promote activation, and neural connectivity in regions associated with social understanding, bodily awareness, and empathy—the insular cortex (Farb, Segal, & Anderson, 2012) and the TPJ (Hölzel et al., 2011). Birnie, Speca, and Carlson (2010) also showed that mindfulness training can increase social connectedness and empathy while decreasing personal distress. Mindfulness training may therefore enhance the processing of emotional expression, a mechanism by which individuals can then connect with others. This was assessed after MBCT training with formerly depressed adults by De Raedt et al. (2012), who found that mindfulness training resulted in more balanced receptiveness to positive and negative facial stimuli, in comparison to controls who also had a history of depression. Such an effect in adolescents who may also be considered vulnerable to depression could lead to enhancing social connectedness and potentially buffer them against mental illness (Donald & Dower, 2002; Music, 2014).

Event‐related brain potentials (ERPs) have several advantages for neurodevelopmental research in an education context: their temporal resolution is high, the recording system is portable thus allowing for testing in schools, and the method is cost‐effective in comparison to fMRI (Sanger & Dorjee, 2015). To our knowledge, however, no neuroscientific research has yet investigated changes in emotion processing with mindfulness training in adolescents, using either fMRI or ERPs. One appropriate ERP marker for detecting such changes is the P300 (particularly the P3b), which indexes task‐related information processing (Sutton, Braren, Zubin, & John, 1965). The P3b has been associated with several brain regions, but evidence suggests a key link with the anterior cingulate cortex (ACC) and TPJ (Kok, 2001; Polich, 2007). The ACC is involved in top‐down regulation of attention and emotion (Bush, Luu, & Posner, 2000), and both brain regions are sensitive to modulation by mindfulness‐based training (Cahn & Polich, 2006; Hölzel et al., 2007; Hölzel et al., 2011). Moreover, the P3b has previously been used as an index of choice in cognitive empathy tasks (e.g., Han, Fan, & Mao, 2008; Ikezawa, Corbera, & Wexler, 2014; Meng et al., 2012). The P3b is also modulated by change in emotive facial processing in adults with depression (Cavanagh & Geisler, 2006). Specifically, P3b amplitude elicited by happy faces was reduced in MDD participants, indicating a limited ability to process positive facial cues. Given the negative correlation between mindfulness and depression documented in previous studies (Deng et al., 2014; Desrosiers et al., 2013), it can be hypothesized that mindfulness training would increase P3b amplitudes to happy faces. Consistent with the predictions of the ECI model of depression and the results of MBCT effects in adults vulnerable to depression (De Raedt et al., 2012), P3b responses to sad faces may equally be modulated, while self‐report measures would show increased well‐being. These predictions would corroborate the pattern of enhanced affective processing, without negative mood induction, which has been observed in previous research (Birnie et al., 2010; Dorjee, Lally, Darral‐Rew, & Thierry, 2015).

The purpose of this study was to investigate longitudinal changes in emotion processing in older secondary school students (16–18 years) after mindfulness training delivered as a module in Personal, Social, and Health Education (PSHE). The evaluations included self‐report questionnaires and P3b modulations recorded in a computerized emotional oddball task in response to affective faces (happy and sad face oddballs—10% of stimuli each type, presented amongst frequent neutral faces—80% of stimuli). The task was based on a previous study by Cavanagh and Geisler (2006) that found reduced P3b response to happy face oddballs and delayed P3b amplitudes to fearful face oddballs in depressed compared to non‐depressed university students. In line with previous findings (Huppert & Johnson, 2010; Metz et al., 2013), we expected that mindfulness training would improve well‐being and mental health in trained adolescents compared to controls. Critically, we expected that P3b responses elicited by emotional stimuli would be significantly increased in the intervention group. This would suggest that mindfulness‐based practice enhances the processing of socially relevant stimuli and healthy emotional exposure, a pattern that contrasts with neurocognitive responses to emotional stimuli in people with depression (Blom et al., 2015; Cavanagh & Geisler, 2006).

## 2 METHODS

### 2.1 Participants

The study was approved by the Ethics Committee in the School of Psychology at Bangor University, prior to participant recruitment. Participants were recruited from four schools in North Wales (UK), two for the training group and two wait‐list controls. The schools were selected based on socioeconomic status and academic attainment and matched on these criteria. All four schools also expressed interest in training their teachers in mindfulness and providing a mindfulness programme to their students as part of regular school curricula. The first two schools to volunteer for participation in the study were assigned to the training group. Sixth form students (16–18 years) from all schools were recruited after presentations describing the study, and sign‐up sheets were placed in sixth‐form common rooms. Participants volunteered for questionnaires plus ERP recordings during an experimental task, or only questionnaires. Those participating in the ERP part of the study were allocated a time‐slot in January–February (pre‐training) and in April–June (post‐training). Participation was open to the entire sixth form for control students, and all those enrolled on the mindfulness course for the training group.

The total sample population was *N* = 48 (21 training group), however class attendance records led to the exclusion of one training group participant who only attended one mindfulness session. In order to compare self‐report and ERP measures, analysis was run only on participants who completed all assessments at both time points. This led to a final sample of 40 students (19 training group, mean [*M*] age 16.8, standard deviation [*SD*] 0.6), as several participants had opted to only complete self‐report measures. Pre‐test differences were assessed using independent *t* tests for age, doctor visits, and sickness absences. A chi‐square analysis was also run for gender, past training relating to stress relief, cognitive skills, and mindfulness experience. No significant pre‐test differences were identified (all *p*s > .05). However, age and gender were marginally significant (*p*s = .06) due to more upper sixth‐form Year 13 participants volunteering in the control group (training group *M* = 16.6, *SD* = 0.6 / control group *M* = 17.0, *SD* = 0.6) and more boys volunteering in the training group. Nevertheless, the difference between 16‐ and 17‐year‐olds in developmental terms is minimal (Palluel, Nougier, & Olivier, 2010; Waxer & Morton, 2011). However, to ensure that the primary mindfulness effects were not impacted, ERP analysis was also run as a hierarchical multiple regression, additionally controlling for age and gender. All other pre‐test differences were non‐significant (all *p*s > .1).

### 2.2 Measures

The Five‐Facet Mindfulness Questionnaire (FFMQ; Baer, Smith, Hopkins, Krietemeyer, & Toney, 2006) assessed whether mindfulness levels increased after training. It has 39 items and five subscales measured on a 5‐point Likert scale: Observing, *“*I pay attention to sensations, such as the wind in my hair or sun on my face”; Describing, “I'm good at finding words to describe my feelings”; Acting with Awareness, “I am easily distracted”; Non‐Judging, “I tell myself I shouldn't be feeling the way I'm feeling”; and Non‐Reacting, “I watch my feelings without getting lost in them”. The final score can be calculated as FFMQ‐Total for each individual, or by subscale, with a higher score indicating a more mindful disposition. The internal consistencies (Cronbach's α) for these facets are good, reported as 0.83 for FFMQ‐O, 0.91 for FFMQ‐D, 0.87 for FFMQ‐AwA, 0.87 for FFMQ‐NJ, and 0.75 for FFMQ‐NR (Baer et al., 2006).

The Perceived Stress Scale (PSS; Cohen, Kamarck, & Mermelstein, 1983) evaluated ability to cope with stress, and has good internal consistency (Cronbach's α = 0.85; Cohen et al., 1983). The PSS has been used previously in mindfulness research, showing reductions in scores in adults (Baer, Carmody, & Hunsinger, 2012) and adolescents (Biegel et al., 2009) after mindfulness training. It consists of 10 items scored on a 5‐point Likert scale, where a higher score indicates more perceived stress during the last month.

The World Health Organization, Well‐Being Index 5‐item version (WHO‐5; WHO Collaborating Centre in Mental Health, 1998) can provide a valid measure of depression levels in adolescents (Blom, Bech, Högberg, Larsson, & Serlachius, 2012), and asks how participants have felt over the past 2 weeks. It uses a 6‐point Likert scale, and phrases questions positively, for example, “I have felt active and vigorous”. It has good internal reliability, with a Cronbach's α of 0.84 (Bech, Olsen, Kjoller, & Rasmussen, 2003).

The Toronto Empathy Questionnaire (TEQ; Spreng, McKinnon, Mar, & Levine, 2009) is a brief self‐report measure created on the basis of a factor‐analysis of longer empathy questionnaires. The measure has been successfully used with adolescents (Barry, Kauten, & Lui, 2014; Brewer & Kerslake, 2015). The TEQ conceptualizes empathy as primarily an emotional process, for example, “When someone else is feeling excited, I tend to get excited too”. It includes 16 questions, eight of which are reverse coded, and scores are measured on a 5‐point Likert scale. The internal reliability with adolescents is good, with Spreng et al. (2009) reporting Cronbach's alpha ranging from 0.85 to 0.87 across three studies.

An acceptability measure was designed for the study, asking mindfulness‐trained students to rate their satisfaction with the mindfulness course and report on home practice. Course enjoyment was measured on a 7‐point Likert scale from 1 (not at all) to 7 (very much) and home practice was measured on a 4‐point Likert scale from 1 (never) to 4 (every day). Class attendance records were accessed via the school.

Participants also provided data pre‐ and post‐training regarding the number of sickness absences, the frequency of visits to their local doctor (GP), and the reason for GP visits. Students were free to refuse any questions.

### 2.3 Emotional oddball paradigm

Happy, sad, and neutral faces from the Karolinska database (Goeleven, De Raedt, Leyman, & Verschuere, 2008) were used in the oddball task. Happy (10%) and sad (10%) faces were the target stimuli, including 15 male and 18 female model face pairs (33 oddball targets per emotion), which participants were instructed to respond to by pressing the spacebar on a computer keyboard, using their dominant hand. The frequent (80%) non‐target faces were two repeated images of one male and one female model with a neutral expression. Data from the Goeleven et al. (2008) validation paper were used to balance stimuli, with analyses of variance (ANOVA) showing no difference in arousal levels between target emotions (*F*(1, 62) = 3.0, *p* = .09), between genders (*F*(1, 62) < 0.1, *p* = .91), and no significant interaction between emotion and gender (*F*(1, 62) = 0.97, *p* = .33). Mean correct emotion identification scores for selected stimuli were > 75%, based on data from Goeleven et al. (2008).

Face stimuli were presented in the centre of the computer monitor for 900 ms, with an inter‐stimulus interval of 750 ms. Participants performed the task in three blocks of 110 trials with the same proportion of the oddball and neutral stimuli in each block, with faces displayed randomly within each block. Block presentation was counterbalanced across participants to control for order effects. The task took 9 minutes to complete, plus breaks between blocks.

### 2.4 Mindfulness‐based programme

An age‐appropriate mindfulness‐based curriculum called ‘.b Foundations’ (MiSP; http://mindfulnessinschools.org/) was delivered over eight 50‐minute sessions plus an initial orientation session. The course was taught by participants’ regular schoolteachers within the PSHE curriculum. For a full description of the implementation model please see Sanger and Dorjee (2016).

### 2.5 Procedures

This study followed a non‐randomized, pre‐post design with wait‐list control group. Participants were tested individually during school hours, scheduled within independent study periods, using a portable EEG system. Quiet testing spaces were provided on school premises. At pre‐test all procedures were explained to participants, and informed consent was obtained before the start of testing. Participants were asked to come to their testing sessions with clean, dry hair and not to use any hair products or conditioner. During the EEG set‐up period students filled in self‐report measures. If these were not completed during set‐up, students took them away in a sealed envelope and were asked to return them on the next school day.

EEG signal was recorded with 36 Ag/AgCl electrodes placed according to the 10–20 standard system, using the right mastoid as the reference site and was re‐referenced offline to the algebraic mean of the right and left mastoid (A1), as is recommended in Luck (2005) and standardly used in practice (e.g., Dorjee et al., 2015; Savill & Thierry, 2011). FPz was used as the system's ground. EEG data were recorded with Neuroscan NuAmps amplifiers with a sampling rate of 1 kHz. Two electrodes placed above and below the right eye monitored ocular movements. In addition, electrodes attached on both forearms recorded heart rate variability; the results of these analyses will be reported elsewhere. Electrode impedance was kept below 7 kΩ. The EEG signal was filtered online with a bandpass filter between 0.01 and 200 Hz, and additional filtering was applied offline using a zero phase shift low‐pass filter with a cut‐off frequency of 30 Hz, and a 48 dB/Oct slope. ERP data were manually cleaned, rejecting motor and irregular ocular artefacts. An algorithm in Neuroscan Edit software was then employed to regress out eye‐blink artefacts, and mathematically remove artefacts using the Gratton, Coles, and Donchin (1983) method. The data were epoched into 1000 ms segments (beginning 100 ms before stimulus onset), and baseline corrected relative to pre‐stimulus activity. Averages for each condition and participant, as well as grand averages across participants for each condition and group, were then computed, considering only correct trials, that is, trials in which happy or sad target faces had been correctly detected. ERPs elicited by neutral faces (standard stimuli) were computed from the last neutral face that preceded an emotional target stimulus. This method of standard ERP calculation ensures that standard stimuli were maximally habituated (i.e., maximally standard in nature) and, by the same token, that trial numbers were comparable between oddball and standard conditions. Mean false alarms to standards during this task were minimal, 0.88 (*SD* 1.1) at pre‐test and 0.85 (*SD* 1.2) at post‐test. Therefore false alarms were not excluded after acquiring only standards that preceded a target trial, as the potential influence of any false alarms that may have been included would be negligible.

### 2.6 Data analysis

**Figure 1:**
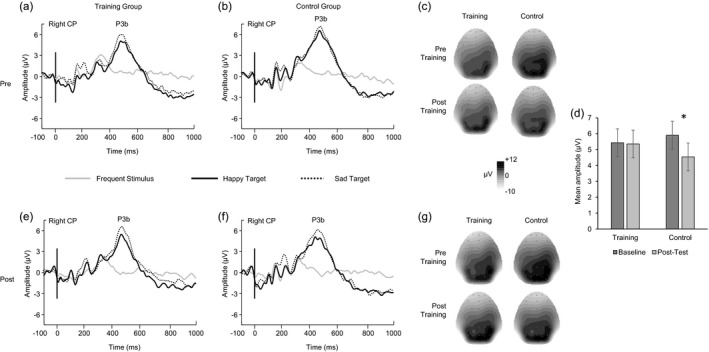
Graphs a, b, e, and f show linear derivations (CP2, CP4, P2, P4) of group average waveforms for each of the three conditions. Topographies c and g show P3b amplitude distribution for happy and sad targets respectively. Graph d represents the P3b mean amplitude change averaged across the three conditions with significant decrease in the control group (*p* < .004)

Pre‐post questionnaire and oddball task performance measures were analysed using a mixed factorial analysis of variance (ANOVA), with time (pre, post) and group (training, control) as factors. Significant interactions were further investigated using paired samples *t* tests. Outliers above and below two standard deviations (*SD*) of the group mean were removed prior to analysis. ERP analysis was conducted over a cluster of centroparietal electrodes identified in a preliminary laterality analysis (CP2, CP4, P2, and P4). Similarly to previous studies investigating P3b responses to emotional stimuli (Keil et al., 2002; Schupp, Junghofer, Weike, & Hamm, 2003; Schupp et al., 2006; Olofsson, Nordin, Sequeira, & Polich, 2008), the distribution was right‐lateralized (Figure 1). An omnibus ANOVA with factors of time, group and condition was then conducted on P3b mean amplitudes. Significant interactions were further investigated using paired sample *t* tests. Greenhouse‐Geisser adjustment for degrees of freedom was applied where applicable. Due to the conflicting evidence in the school‐based literature regarding the impact of mindfulness training engagement on outcomes (Foret et al, 2012; Huppert & Johnson, 2010), we ran correlation analyses to assess any moderating effects of course attendance, satisfaction, and home practice in the training group. In addition, potential relationships between differential P3b amplitudes (deviant minus‐standard) and empathy scores across groups were studied. This approach to correlations with self‐report measures was also used by Ikezawa et al. (2014) and Fan and Han (2008). Lastly, a hierarchical multiple regression using post‐training average P3b mean amplitudes as the dependent variable for all task conditions, controlling for pre‐training mean amplitudes and group, then age, then gender, examined whether the marginal group differences in age and gender impacted on the main ERP findings.

## 3 RESULTS

### 3.1 Five‐Facet Mindfulness Questionnaire

The FFMQ had an acceptable level of internal reliability (Cronbach's α) within this sample at pre‐test, 0.82 for FFMQ‐Total, 0.76 for FFMQ‐O, 0.86 for FFMQ‐D, 0.83 for FFMQ‐AwA, 0.87 for FFMQ‐NJ, and 0.68 for FFMQ‐NR. Mixed factorial ANOVA results for FFMQ‐Total did not reveal any significant effects (all *p*s > .1). Subscales were also analysed, but no significant effects were found (all *p*s > .1).

### 3.2 Perceived‐Stress Scale

Pre‐test reliability for the PSS was good (α = 0.83). Two participants did not provide responses on this measure, reducing the sample to *n* = 38. There was a marginal main effect of time (*F*(1, 36) = 3.6, *p* = .07) but no significant group effect (*F*(1, 36) = .2, *p* = .66) or interaction (*F*(1, 36) = 1.5, *p* = .23). A significant positive correlation was found between PSS change scores and self‐reported enjoyment of the mindfulness course, suggesting that participants in the training group who reported more enjoyment of the course actually increased in perceived stress (*r* = .65, *p* = .004). Interestingly, post‐hoc analysis found that perceived stress change pre‐post did not correlate with change in well‐being scores in the training group (*r* = −.08, *p* = .78), while a strong relationship between these variables was found in the control group (*r* = −.76, *p* < .001).

### 3.3 WHO‐5 well‐being index

The WHO‐5 was reliable within this sample (α = 0.79). Five participants did not contribute to this measure, reducing the sample to *n* = 35. The mixed factorial ANOVA revealed a significant main effect of time (*F*(1, 33) = 12.9, *p* = .001, *ƞ* ^*2*^ = .26), with all participants’ well‐being increasing by post‐test. There was a non‐significant effect of group (*F*(1, 33) < .1, *p* = .81), and marginally significant time by group interaction (*F*(1, 33) = 3.1, *p* = .08, *ƞ* ^*2*^ = .06). As the interaction was in the predicted direction, follow‐up paired samples *t* tests were conducted, showing the trend to be due to a significant increase in self‐reported well‐being in the training group (*t*(15) = −4.3, *p* = .001, *d* = 1.1), whilst no significant changes were observed in the control group (*t*(18) = −1.2, *p* = .24). The between‐group comparison at the post‐test was not significant (*p* > .1)

### 3.4 TEQ empathy questionnaire

The reliability of this measure at pre‐test was good (α = 0.84). Four participants did not provide responses, reducing the sample to *n* = 36. The ANOVA showed a significant main effect of time (*F*(1, 34) = 10.1, *p* = .003, *ƞ* ^*2*^ = .23), with participants’ empathy reducing pre‐post, and a significant main effect of group (*F*(1, 34) = 8.8, *p* = .006, *ƞ* ^*2*^ = .21), indicating that the training group were less empathetic on average. The interaction effect was non‐significant (*F*(1, 34) = .3, *p* = .61). However, changes in this measure correlated positively with course attendance (*r* = .66, *p* = .006) and marginally with home practice (*r* = .49, *p* = .06), suggesting that those who attended more of the mindfulness course and practised more displayed larger increases in empathy.

### 3.5 Health measures

ANOVAs revealed no change in absenteeism over time (*F*(1, 38) = .6, *p* = .45), between group (*F*(1, 38) = 1.3, *p* = .25) or an interaction (*F*(1, 38) = .9, *p* = .35). GP visits were also not affected by time (*F*(1, 38) = .6, *p* = .44) or group differences (*F*(1, 38) = 1.2, *p* = .28), the time by group interaction was marginally significant (*F*(1, 38) = 3.0, *p* = .09, *ƞ* ^*2*^ = .07). To investigate the possibility of differential effects for visits due to physical and mental health reasons, GP visits were further broken down accordingly (e.g., asthma and stress, respectively). For mental health‐related visits only, the ANOVA showed no change over time (*F*(1, 38) = .3, *p* = .58) or group (*F*(1, 38) = .7, *p* = .42), but there was a significant time by group interaction (*F*(1, 38) = 5.0, *p* = .03, *ƞ* ^*2*^ = .12). However, follow‐up paired samples *t* tests revealed only trends towards significance, with some reduction in the training group (*t*(18) = 1.7, *p* = .11, *d* = .39) and a non‐significant increase in GP visits over time in the control group (*t*(20) = −1.5, *p* = .16, *d* = .32). Nevertheless the effect sizes suggest a small but meaningful association between mindfulness practice and health, and, given the short time‐scale this was measured over (8 weeks), it merits consideration. On visual inspection there appeared to be pre‐test group differences on GP visits (see Table 1). However, independent t‐tests confirmed that these were only marginally significant at pre‐test for general and specifically mental health visits (*p*s > .05). This was most likely due to the non‐randomized nature of participant recruitment.

**Table 1:** Pre to post self‐report measure changes in training and control groups (* < .05; ** < .01; ~ = trend towards significance in follow‐up *t* test analysis)

Questionnaire means (*SD*)	Pre‐training group	Post‐training group	Pre‐control group	Post‐control group
FFMQ‐Total	119.9 (15.9)	120.4 (12.7)	121.1 (17.1)	122.3 (20.6)
FFMQ‐O	24.4 (6.3)	24.1 (5.3)	24.6 (5.2)	23.6 (5.3)
FFMQ‐D	26.1 (5.8)	24.6 (4.3)	26.0 (6.3)	26.4 (5.7)
FFMQ‐AwA	24.1 (5.4)	23.3 (4.2)	24.2 (6.9)	23.8 (6.5)
FFMQ‐NJ	25.8 (6.4)	27.5 (5.9)	26.0 (6.6)	27.7 (6.5)
FFMQ‐NR	19.6 (3.4)	20.7 (3.5)	20.4 (4.4)	20.9 (4.5)
PSS	20.1 (6.7)	19.8 (4.9)	22.5 (6.7)	19.7 (6.0)
WHO‐5	48.0 (16.7)	64.8 (20.4)	55.6 (19.6)	61.1 (16.9)
TEQ	43.1 (10.8)	39.8 (8.7)	50.5 (7.1)	48.1 (7.0)
Health‐Absenteeism	3.5 (3.7)	3.4 (3.8)	4.9 (5.5)	5.9 (7.5)
Health‐GP Visits	1.1 (2.4)	0.5 (0.7)	0.3 (0.8)	0.5 (0.7)
Health‐Psychological GP Visits	0.4 (1.0)	0.1 (0.2)	0 (0)	0.2 (0.6)

### 3.6 Task performance

Mixed factorial ANOVAs assessed oddball task performance. There was a significant main effect of time on accuracy to happy target faces (*F*(1, 38) = 4.8, *p* = .04, *ƞ* ^*2*^ = .11), but no significant main effect of group (*F*(1, 38) < .1, *p* = .88), and no significant interaction (*F*(1, 38) = .5, *p* = .48), suggesting that all participants became more accurate by post‐test. A marginally significant positive correlation was found between mindfulness course enjoyment and improved target accuracy to happy faces (*r* = .40, *p* = .07). No significant effects of accuracy were found for sad target faces (all *p*s > .1). In terms of response time (RT), all target effects were non‐significant (*p*s > .1) except a marginal group effect for sad targets (*F*(1, 38) 3.29, *p* = .08, *ƞ* ^*2*^ = .08), suggesting a small increase in RT across groups. False alarms to standard stimuli were minimal, 0.88 (*SD* 1.1) at pre‐test and 0.85 (*SD* 1.2) at post‐test over the 267 standard trials. The ANOVA showed no significant main effects or interactions (all *p*s > .1).

### 3.7 P3b ERP analysis

A 2 (time: pre, post) × 3 (condition: happy, sad, standard) × 2 (group: training, control) ANOVA assessed any variation in trial numbers included in ERP analysis across conditions and groups. There were no significant main effects of time or group (*p*s > .1), but there was a significant main effect of condition (*F*(2, 76) = 2503.7, *p* < .001, *ƞ* ^*2*^ = .95). This was further explored using simple contrasts showing that this difference was significant between standard and oddball conditions (*p* < .001), and marginally significant between positive and negative emotional targets (*p* = .06). Thus, the rejection rates were significantly higher for standards than oddballs and marginally higher for sad than happy faces. As shown in Table 2, the difference between the happy and sad face oddballs only amounted to about one in 32 trials. There were no rejection rate differences for any of the conditions between groups at either baseline or post‐test.

**Table 2:** Means and standard deviations (*SD*) for average number of trials included in ERP analysis across groups and conditions

	Mean (*SD*) trials per condition for ERP analysis					
	Pre‐happy	Pre‐sad	Pre‐standard neutral	Post‐happy	Post‐sad	Post‐standard neutral
**Training group**	32.1 (.8)	31.6 (1.6)	52.5 (3.5)	31.2 (1.9)	31.1 (1.9)	50.5 (4.0)
**Control group**	32.0 (1.5)	31.4 (2.0)	51.4 (4.4)	31.6 (1.3)	31.2 (1.9)	51.8 (3.3)

The time window for the P3b analyses (420–520 ms) was determined by inspection of the global field power calculated across the scalp, which provided a synthetic view of key change points of power with time. First, the distribution of the P3b was assessed by an analysis of the pre‐test mean amplitudes using combined averages for the two oddball conditions and groups, thereby reducing the number of ANOVA factors and thus minimizing the likelihood of spurious effects (Luck & Gaspelin, 2017). The resulting 2 × 4 ANOVA with factors of laterality (left, right) and electrode (left: CP1, CP3, P1, P3; right: CP2, CP4, P2, P4) revealed a significant main effect of laterality (*F*(1, 39) = 15.11, *p* < .001, *ƞ* ^*2*^ = .27) with greater mean amplitudes on the right (left *M* = 6.36 μV, right *M* = 7.71 μV, Figure 1). There was also a significant main effect of electrode (*F*(1.70, 66.17) = 8.01, *p* < .002, *ƞ* ^*2*^ = .17) and a significant laterality‐by‐electrode interaction (*F*(2.26, 88.19) = 6.28, *p* < .003, *ƞ* ^*2*^ = .14). Therefore, further analyses including time and group factors focused on the linear derivation of the right–sided cluster of four electrodes (CP2, CP4, P2, P4), thus removing the electrode factor from further analyses as recommended by Luck and Gaspelin (2017).

A 2 × 2 × 3 ANOVA with factors of time (pre, post), group (training, control) and condition (happy, sad, neutral) assessed possible changes across time in mean amplitudes of the P3b (Figure 1). There was a significant main effect of time (*F*(1, 38) = 5.88, *p* < .03, *ƞ* ^*2*^ = .13) which indicated a significant decrease in mean amplitudes from pre (*M* = 5.68 μV) to post‐test (*M* = 4.93 μV). There was no significant main effect of group (*p* > .1). The main effect of condition was significant (*F*(1.63, 61.89) = 88.74, *p* < .001, *ƞ* ^*2*^ = .70) with sad faces eliciting the largest mean amplitudes (*M* = 8.07 μV), followed by happy faces (*M* = 6.75 μV) and neutral faces (*M* = 1.11 μV). Importantly, the time‐by‐group interaction was also significant (*F*(1, 38) = 4.73, *p* < .04, *ƞ* ^*2*^ = 0.11). Follow‐up analyses revealed that the mean amplitudes across conditions significantly decreased in the control group over time (*t*(20) = 3.32, *p* < .004, *d* = .34), whereas the training group did not show any significant change from pre to post‐test (*p* > .1). None of the remaining interactions were significant (all *p*s > .1). The ANOVA for latency revealed no significant effects (all *p*s > .1).

**Figure 2:**
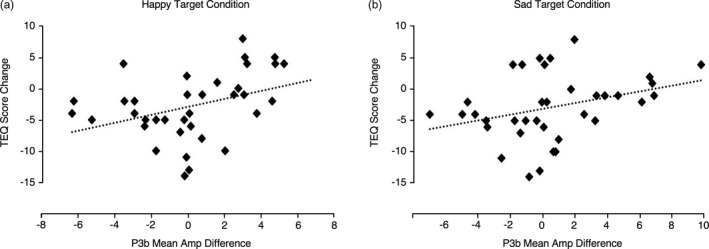
Correlation plots show the significant positive relationship between P3b mean amplitude changes (post‐training minus pre‐training in both groups combined to happy (a; *p* = .03) and sad (b; *p* = .05) target stimuli with neutral condition subtracted) and change (post minus pre) in TEQ empathy questionnaire scores

To further investigate the relationship between P3b mean amplitude modulations (post‐ versus pre‐training) and changes in measures of empathy (post minus pre TEQ scores), mean P3b amplitudes to neutral faces were subtracted from the mean P3b amplitudes to happy and sad face oddballs, respectively. This correlation was positive for both happy and sad faces (happy: *r* = .37, *p* = .03; sad: *r* = .33, *p* = .05; Figure 2).

## 4 DISCUSSION

We studied emotional face processing before and after a school‐based mindfulness programme in adolescents. Mindfulness training was associated with maintained P3b mean amplitudes to correctly identified happy and sad target faces in an oddball task involving neutral faces as standard stimuli, whereas P3b amplitude was overall reduced over time in wait‐list controls. There were no between‐group effects on response time or accuracy, but mindfulness course satisfaction positively correlated with improved accuracy for happy faces. Training‐based improvements were also noted in self‐reported well‐being and health measures, with significant increases on the WHO‐5 well‐being index and trends towards fewer mental health‐related GP visits. Although mindfulness training did not impact TEQ scores overall, course attendance and home practice both positively correlated with empathy change. Overall, this study demonstrated that a mindfulness‐based PSHE module delivered by internal schoolteachers can positively impact students’ processing of faces and well‐being.

Modulation in P3b responses to happy and sad faces also correlated with changes in empathy. This suggests that mindfulness practice may help sustain attentional focus on socially relevant affective stimuli, overriding typical stimulus habituation (Geisler & Polich, 1994; Ravden & Polich, 1998), and potentially indexing heightened empathy. The finding of stable P3b amplitudes elicited by face stimuli, along with self‐reported well‐being increases, also aligns with our initial predictions and is consistent with previous research, showing that adolescents with MDD tend to show reduced levels of neural activity when processing sad faces (Blom et al., 2015). In addition, research supporting the ECI model of depression showed that many adults with MDD have a general impairment in processing affective stimuli manifesting through dampened responses (Bylsma et al., 2008). In contrast, mindfulness training has been shown to enhance brain activity and mood in adults (Bostanov, Keune, Kotchoubey, & Hautzinger, 2012; Williams et al., 2014). This study was the first to demonstrate the modulation of depression‐related ERP markers of emotion processing in adolescents. Tentatively, we can interpret the findings as suggesting that mindfulness practice, which encourages curiosity and exposure to emotion without judgement or reactivity, can help maintain attention on socially relevant stimuli, in comparison to habituation patterns observed in controls. We note that attention shifts indexed by P3b amplitude were observed irrespective of emotional valence and thus could be underpinned by more generic attention modulation resulting from mindfulness training.

The self‐report findings support previous research on school‐based mindfulness training, showing a significant increase in well‐being (Huppert & Johnson, 2010; Metz et al., 2013). The inclusion of health‐related data adds to insights from previous school‐based interventions, suggesting that mindfulness training may reduce adolescents’ needs to seek mental health advice. Marginal decreases in GP visits for psychological reasons (e.g., stress, trouble sleeping) were found in the training group, as control participants reported slight increases. This divergent pattern of GP visits was supported by a small to moderate effect size, which is important to examine given the limited sample size and timeframe. The timing of data collection may be relevant here, as students were preparing for summer exams and the potential for stress and anxiety would have been high. Thus, mindfulness practice may have had a buffering effect on psychological well‐being, manifesting in less need to seek help during a challenging period.

No changes in perceived stress were found between groups although previous school‐based and adult intervention studies have found reductions (Baer et al., 2012; Biegel et al., 2009; Metz et al., 2013; Shapiro, Brown, & Biegel, 2007), but the current data showed a positive correlation between perceived stress change scores and mindfulness course satisfaction. Post‐hoc, we explored the relationship between PSS and WHO‐5 fluctuations. Whilst self‐reported change on these measures was strongly correlated in controls, there was no such relationship in training participants. Mindfulness is the practice of attending to the present moment with curiosity and acceptance, allowing people to become aware of their experience without reactivity (Kumar, Feldman, & Hayes, 2008; Goldin & Gross, 2010). These data would suggest that mindfulness practice enhanced students’ affective awareness without impairing well‐being, an emotion regulation strategy previously found in adults with anxiety problems learning mindfulness (Goldin & Gross, 2010).

Moreover, no pre‐post change was seen in empathy scores, but within the training group both course attendance and home practice positively correlated with increased empathy. We speculate that greater engagement with mindfulness practice could be required for self‐reported empathy change. Interestingly, P3b mean amplitude to targets, which were significantly modulated by mindfulness training, also correlated with empathy scores. It may be that ERP measures operate as a more sensitive measure of changes in social cognition and it is only with prolonged, or potentially more experienced guidance in, mindfulness practice that self‐reported changes in empathy would be found.

The lack of an active control group is a limitation of this study, as the experience of a new curriculum in school could have impacted students. However, the PSHE curriculum in Wales for 16+ students does cover morality, well‐being, spirituality, and metacognition (Dept for Children, Education, Life‐long Learning and Skills, 2008) and students are taught new topics throughout the school year. A schoolteacher specializing in a different subject teaches PSHE, and it has an atypical set‐up involving active discussion and group work in a more relaxed environment than typical lessons. So, although novelty was not controlled for, in many other respects the standard PSHE curriculum can be considered a suitable active control for a mindfulness‐based course.

It should also be acknowledged that in this study it was the first time that the teachers who were involved had delivered a mindfulness‐based curriculum, and after only 9 months of personal practice. While all teachers were considered ready by an experienced mindfulness trainer (in terms of readiness to deliver the programme), they remained relatively inexperienced. This limitation of our study can also be construed as reflecting the conditions of implementation to be expected in the real world.

The positive correlation between mindfulness course satisfaction and improved target accuracy to happy faces could have resulted from increased performance motivation. However, three other results go against motivation bias. First, there was no difference in RT and target accuracy between groups at pre‐ or post‐test. Second, course satisfaction and perceived stress correlated significantly. Third, the marginal decrease over time in GP visits was a naturalistic behavioural effect, not a performance‐based outcome, and is unlikely to have been impacted by motivation bias. Nevertheless, future studies should take motivational bias into consideration and include manipulations that can reduce its effects, such as an active control group with a second intervention.

The limited sample size for self‐report measures must be considered. Although using the same sample for self‐report and ERP measures ensures a more accurate comparison between assessments, it constrains the likelihood of finding significant differences between groups. However, effect sizes arguably better reflect the impact of mindfulness training on adolescents, as these are not so constrained by sample size (Cumming, 2012). Another limitation was the lack of follow‐up results in this study, which is a common problem in neuroscientific intervention studies, for logistical and cost‐related reasons. We hope that future work will build on these initial findings and explore the long‐term effects of mindfulness training in adolescents. Of particular interest could be improvements in self‐reported well‐being and empathy after continued practice, as well as sustained changes in ERP indices of emotional processing predictive of anxiety and depression. These should provide more conclusive evidence of possible long‐term effects of mindfulness training delivered within a school context. Future investigations should map the trajectory of how empathy might develop with prolonged mindfulness practice, or how learning from teachers with more experience might reflect on outcomes for their students.

### 4.1 Conclusion

To our knowledge, this non‐randomized controlled study was the first neurocognitive investigation of longitudinal modulation in face processing in adolescents resulting from mindfulness training delivered by schoolteachers as part of a regular school curriculum. The results show that mindfulness training can maintain participants’ attention and associated exposure to socially relevant stimuli, while improving self‐reported well‐being. Mindfulness practice may also have a buffering effect on stress‐related illness, as suggested by the small modulations in GP visits over 8 weeks. Overall, these findings suggest that mindfulness training delivered as part of the school curriculum might be effective in improving the well‐being of older school‐aged students during a period of heightened stress and depression vulnerability. The study also highlights the potential of neuroscientific methods in contributing to our understanding of mindfulness effects in education.

## References

[desc12646-bib-0001] Baer, R.A. , Carmody, J. , & Hunsinger, M. (2012). Weekly change in mindfulness and perceived stress in a mindfulness‐based stress reduction program. Journal of Clinical Psychology, 68, 755–765. 22623334 10.1002/jclp.21865

[desc12646-bib-0002] Baer, R.A. , Smith, G.T. , Hopkins, J. , Krietemeyer, J. , & Toney, L. (2006). Using self‐report assessment methods to explore facets of mindfulness. Assessment, 13, 27–45. 16443717 10.1177/1073191105283504

[desc12646-bib-0003] Barry, C.T. , Kauten, R.L. , & Lui, J.H. (2014). Self‐perceptions of empathy and social support as potential moderators in the relation between adolescent narcissism and aggression. Individual Differences Research, 12, 170–179.

[desc12646-bib-0004] Bech, P. , Olsen, L.R. , Kjoller, M. , & Rasmussen, N.K. (2003). Measuring well‐being rather than the absence of distress symptoms: A comparison of the SF‐36 Mental Health subscale and the WHO‐Five well‐being scale. International Journal of Methods in Psychiatric Research, 12, 85–91. 12830302 10.1002/mpr.145 PMC6878541

[desc12646-bib-0005] Biegel, G.M. , Brown, K.W. , Shapiro, S.L. , & Schubert, C.M. (2009). Mindfulness‐based stress reduction for the treatment of adolescent psychiatric outpatients: A randomized clinical trial. Journal of Consulting and Clinical Psychology, 77, 855–866. 19803566 10.1037/a0016241

[desc12646-bib-0006] Birnie, K. , Speca, M. , & Carlson, L.E. (2010). Exploring self‐compassion and empathy in the context of mindfulness‐based stress reduction (MBSR). Stress and Health, 26, 359–371.

[desc12646-bib-0007] Block‐Lerner, J. , Adair, C. , Plumb, J.C. , Rhatigan, D.L. , & Orsillo, S.M. (2007). The case for Mindfulness‐based approaches in the cultivation of empathy: Does nonjudgmental, present‐moment awareness increase capacity for perspective‐taking and empathic concern? Journal of Marital and Family Therapy, 33, 501–516. 17935532 10.1111/j.1752-0606.2007.00034.x

[desc12646-bib-0008] Blom, E.H. , Bech, P. , Högberg, G. , Larsson, J.O. , & Serlachius, E. (2012). Screening for depressed mood in an adolescent psychiatric context by brief self‐assessment scales: Testing psychometric validity of WHO‐5 and BDI‐6 indices by latent trait analyses. Health and Quality of Life Outcomes, 10, 149. 23227908 10.1186/1477-7525-10-149 PMC3575311

[desc12646-bib-0009] Blom, E.H. , Connolly, C.G. , Ho, T.C. , LeWinn, K.Z. , Mobayed, N. , Han, L. , … Yang, T.T. (2015). Altered insular activation and increased insular functional connectivity during sad and happy face processing in adolescent major depressive disorder. Journal of Affective Disorders, 178, 215–223. 25827506 10.1016/j.jad.2015.03.012 PMC4412607

[desc12646-bib-0010] Bostanov, V. , Keune, P.M. , Kotchoubey, B. , & Hautzinger, M. (2012). Event‐related brain potentials reflect increased concentration ability after mindfulness‐based cognitive therapy for depression: A randomized clinical trial. Psychiatry Research, 199, 174–180. 22771173 10.1016/j.psychres.2012.05.031

[desc12646-bib-0011] Brewer, G. , & Kerslake, J. (2015). Cyberbullying, self‐esteem, empathy and loneliness. Computers in Human Behavior, 48, 255–260.

[desc12646-bib-0012] Bush, G. , Luu, P. , & Posner, M.I. (2000). Cognitive and emotional influences in anterior cingulate cortex. Trends in Cognitive Sciences, 4, 215–222. 10827444 10.1016/s1364-6613(00)01483-2

[desc12646-bib-0013] Bylsma, L.M. , Morris, B.H. , & Rottenberg, J. (2008). A meta‐analysis of emotional reactivity in major depressive disorder. Clinical Psychology Review, 28, 676–691. 18006196 10.1016/j.cpr.2007.10.001

[desc12646-bib-0014] Cahn, B.R. , & Polich, J. (2006). Meditation states and traits: EEG, ERP, and neuroimaging studies. Psychological Bulletin, 132, 180–211. 16536641 10.1037/0033-2909.132.2.180

[desc12646-bib-0015] Carlson, L.E. , Speca, M. , Patel, K.D. , & Goodey, E. (2003). Mindfulness‐based stress reduction in relation to quality of life, mood, symptoms of stress, and immune parameters in breast and prostate cancer outpatients. Psychosomatic Medicine, 65, 571–581. 12883107 10.1097/01.psy.0000074003.35911.41

[desc12646-bib-0016] Cavanagh, J. , & Geisler, M.W. (2006). Mood effects on the ERP processing of emotional intensity in faces: A P3 investigation with depressed students. International Journal of Psychophysiology, 60, 27–33. 15963586 10.1016/j.ijpsycho.2005.04.005

[desc12646-bib-0017] Cohen, S. , Kamarck, T. , & Mermelstein, R. (1983). A global measure of perceived stress. Journal of Health and Social Behavior, 24, 386–396. 6668417

[desc12646-bib-0018] Cumming, G. (2012). Understanding the new statistics: Effect sizes, confidence intervals, and meta‐analysis. New York: Routledge.

[desc12646-bib-0019] Deng, Y.Q. , Li, S. , & Tang, Y.Y. (2014). The relationship between wandering mind, depression and mindfulness. Mindfulness, 5, 124–128.

[desc12646-bib-0020] Department of Children, Education, Life‐Long Learning and Skills. Welsh Assembly Government . (2008). Personal and social education framework for 7 to 19‐year‐olds in Wales. Retrieved from: http://learning.gov.wales/resources/browse-all/personal-and-social-education/?lang=en

[desc12646-bib-0021] De Raedt, R. , Baert, S. , Demeyer, I. , Goeleven, E. , Raes, A. , Visser, A. , … Speckens, A. (2012). Changes in attentional processing of emotional information following mindfulness‐based cognitive therapy in people with a history of depression: Towards an open attention for all emotional experiences. Cognitive Therapy and Research, 36, 612–620.

[desc12646-bib-0022] Desrosiers, A. , Klemanski, D.H. , & Nolen‐Hoeksema, S. (2013). Mapping mindfulness facets onto dimensions of anxiety and depression. Behavior Therapy, 44, 373–384. 23768665 10.1016/j.beth.2013.02.001 PMC4012250

[desc12646-bib-0023] Donald, M. , & Dower, J. (2002). Risk and protective factors for depressive symptomatology among a community sample of adolescents and young adults. Australian and New Zealand Journal of Public Health, 26, 555–562. 12530801 10.1111/j.1467-842x.2002.tb00366.x

[desc12646-bib-0024] Dorjee, D. , Lally, N. , Darrall‐Rew, J. , & Thierry, G. (2015). Dispositional mindfulness and semantic integration of emotional words: Evidence from event‐related brain potentials. Neuroscience Research, 97, 45–51. 25797495 10.1016/j.neures.2015.03.002

[desc12646-bib-0025] Eddy, M.D. , Brunyé, T.T. , Tower‐Richardi, S. , Mahoney, C.R. , & Taylor, H.A. (2015). The effect of a brief mindfulness induction on processing of emotional images: An ERP study. Frontiers in Psychology, 6, 1391. 26441766 10.3389/fpsyg.2015.01391 PMC4566001

[desc12646-bib-0026] Ernst, M. , Pine, D.S. , & Hardin, M. (2006). Triadic model of the neurobiology of motivated behavior in adolescence. Psychological Medicine, 36, 299–312. 16472412 10.1017/S0033291705005891 PMC2733162

[desc12646-bib-0027] Fan, Y. , & Han, S. (2008). Temporal dynamic of neural mechanisms involved in empathy for pain: An event‐related brain potential study. Neuropsychologia, 46, 160–173. 17825852 10.1016/j.neuropsychologia.2007.07.023

[desc12646-bib-0028] Farb, N.A. , Segal, Z.V. , & Anderson, A.K. (2012). Mindfulness meditation training alters cortical representations of interoceptive attention. Social Cognitive and Affective Neuroscience, 8, 15–26. 22689216 10.1093/scan/nss066 PMC3541492

[desc12646-bib-0029] Foret, M.M. , Scult, M. , Wilcher, M. , Chudnofsky, R. , Malloy, L. , Hasheminejad, N. , & Park, E.R. (2012). Integrating a relaxation response‐based curriculum into a public high school in Massachusetts. Journal of Adolescence, 35, 325–332. 21893336 10.1016/j.adolescence.2011.08.008

[desc12646-bib-0030] Geisler, M.W. , & Polich, J. (1994). P300 habituation from visual stimuli? Physiology and Behaviour, 56, 511–516. 10.1016/0031-9384(94)90294-1 7972401

[desc12646-bib-0031] Goeleven, E. , De Raedt, R. , Leyman, L. , & Verschuere, B. (2008). The Karolinska directed emotional faces: A validation study. Cognition and Emotion, 22, 1094–1118.

[desc12646-bib-0032] Goldin, P.R. , & Gross, J.J. (2010). Effects of mindfulness‐based stress reduction (MBSR) on emotion regulation in social anxiety disorder. Emotion, 10, 83–91. 20141305 10.1037/a0018441 PMC4203918

[desc12646-bib-0033] Gotlib, I.H. , & Neubauer, D.L. (2000). Information‐processing approaches to the study of cognitive biases in depression In Johnson S.L., Hayes A.M., Field T.M., Schneiderman N., & McCabe P.M. (Eds.), Stress, coping, and depression (pp. 117–143). Hillsdae, NJ: Lawrence Erlbaum Associates.

[desc12646-bib-0034] Gratton, G. , Coles, M.G.H. , & Donchin, E. (1983). A new method for off‐line removal of ocular artefact. Electroencephalography and Clinical Neurophysiology, 55, 468–484. 6187540 10.1016/0013-4694(83)90135-9

[desc12646-bib-0035] Han, S. , Fan, Y. , & Mao, L. (2008). Gender difference in empathy for pain: An electrophysiological investigation. Brain Research, 1196, 85–93. 18221733 10.1016/j.brainres.2007.12.062

[desc12646-bib-0036] Hargus, E. , Crane, C. , Barnhofer, T. , & Williams, J.M.G. (2010). Effects of mindfulness on meta‐awareness and specificity of describing prodromal symptoms in suicidal depression. Emotion, 10, 34–42. 20141300 10.1037/a0016825 PMC3933215

[desc12646-bib-0037] Hölzel, B.K. , Carmody, J. , Vangel, M. , Congleton, C. , Yerramsetti, S.M. , Gard, T. , & Lazar, S.W. (2011). Mindfulness practice leads to increases in regional brain gray matter density. Psychiatry Research: Neuroimaging, 191, 36–43. 10.1016/j.pscychresns.2010.08.006 PMC3004979 21071182

[desc12646-bib-0038] Hölzel, B.K. , Ott, U. , Hempel, H. , Hackl, A. , Wolf, K. , Stark, R. , & Vaitl, D. (2007). Differential engagement of anterior cingulate and adjacent medial frontal cortex in adept meditators and non‐meditators. Neuroscience Letters, 421, 16–21. 17548160 10.1016/j.neulet.2007.04.074

[desc12646-bib-0039] Huppert, F.A. , & Johnson, D.M. (2010). A controlled trial of mindfulness training in schools: The importance of practice for an impact on well‐being. Journal of Positive Psychology, 5, 264–274.

[desc12646-bib-0040] Ikezawa, S. , Corbera, S. , & Wexler, B.E. (2014). Emotion self‐regulation and empathy depend upon longer stimulus exposure. Social Cognitive and Affective Neuroscience, 9, 1561–1568. 24064924 10.1093/scan/nst148 PMC4187273

[desc12646-bib-0041] Kabat‐Zinn, J. (1994). Wherever you go, there you are: Mindfulness meditation in everyday life. New York: Hyperion.

[desc12646-bib-0042] Keil, A. , Bradley, M.M. , Hauk, O. , Rockstroh, B. , Elbert, T. , & Lang, P.J. (2002). Large‐scale neural correlates of affective picture processing. Psychophysiology, 39, 641–649. 12236331 10.1017/S0048577202394162

[desc12646-bib-0043] Kok, A. (2001). On the utility of P3 amplitude as a measure of processing capacity. Psychophysiology, 38, 557–577. 11352145 10.1017/s0048577201990559

[desc12646-bib-0044] Kumar, S. , Feldman, G. , & Hayes, A. (2008). Changes in mindfulness and emotion regulation in an exposure‐based cognitive therapy for depression. Cognitive Therapy and Research, 32, 734–744.

[desc12646-bib-0045] Kuyken, W. , Weare, K. , Ukoumunne, O.C. , Vicary, R. , Motton, N. , Burnett, R. , … Huppert, F. (2013). Effectiveness of the Mindfulness in Schools Programme: Non‐randomised controlled feasibility study. British Journal of Psychiatry, 203, 126–131. 23787061 10.1192/bjp.bp.113.126649

[desc12646-bib-0046] Luck, S.J. (2005). Ten simple rules for designing ERP experiments In Handy Todd C. (Ed.), Event‐related potentials: A methods handbook (pp. 17–32). Cambridge, MA: MIT Press.

[desc12646-bib-0047] Luck, S.J. , & Gaspelin, N. (2017). How to get statistically significant effects in any ERP experiment (and why you shouldn't). Psychophysiology, 54, 146–157. 28000253 10.1111/psyp.12639 PMC5178877

[desc12646-bib-0048] Meng, J. , Hu, L. , Shen, L. , Yang, Z. , Chen, H. , Huang, X. , & Jackson, T. (2012). Emotional primes modulate the responses to others’ pain: An ERP study. Experimental Brain Research, 220, 277–286. 22695721 10.1007/s00221-012-3136-2

[desc12646-bib-0049] Metz, S.M. , Frank, J.L. , Reibel, D. , Cantrell, T. , Sanders, R. , & Broderick, P.C. (2013). The effectiveness of the learning to BREATHE program on adolescent emotion regulation. Research in Human Development, 10, 252–272.

[desc12646-bib-0050] Music, G. (2014). The buzz trap: Speeded‐up lives, distractedness, impulsiveness and decreasing empathy. Psychodynamic Practice, 20, 228–249.

[desc12646-bib-0051] NICE (National Institute for Health and Care Excellence) (2015), Depression in children and young people: Identification and management in primary, community and secondary care, NICE Clinical Guideline 28, ISBN 978‐1‐4731‐1041‐0.

[desc12646-bib-0052] Olofsson, J.K. , Nordin, S. , Sequeira, H. , & Polich, J. (2008). Affective picture processing: An integrative review of ERP findings. Biological Psychology, 77, 247–265. 18164800 10.1016/j.biopsycho.2007.11.006 PMC2443061

[desc12646-bib-0053] Ortner, C.N. , Kilner, S.J. , & Zelazo, P.D. (2007). Mindfulness meditation and reduced emotional interference on a cognitive task. Motivation and Emotion, 31, 271–283.

[desc12646-bib-0054] Palluel, E. , Nougier, V. , & Olivier, I. (2010). Postural control and attentional demand during adolescence. Brain Research, 1358, 151–159. 20735993 10.1016/j.brainres.2010.08.051

[desc12646-bib-0055] Polich, J. (2007). Updating P300: An integrative theory of P3a and P3b. Clinical Neurophysiology, 118, 2128–2148. 17573239 10.1016/j.clinph.2007.04.019 PMC2715154

[desc12646-bib-0056] Raes, F. , Griffith, J.W. , Van der Gucht, K. , & Williams, J.M.G. (2014). School‐based prevention and reduction of depression in adolescents: A cluster‐randomized controlled trial of a mindfulness group program. Mindfulness, 5, 477–486.

[desc12646-bib-0057] Ravden, D. , & Polich, J. (1998). Habituation of P300 from visual stimuli. International Journal of Psychophysiology, 30, 359–365. 9834892 10.1016/s0167-8760(98)00039-7

[desc12646-bib-0058] Rottenberg, J. , Gross, J.J. , & Gotlib, I.H. (2005). Emotion context insensitivity in major depressive disorder. Journal of Abnormal Psychology, 114, 627–639. 16351385 10.1037/0021-843X.114.4.627

[desc12646-bib-0059] Sanger, K.L. , & Dorjee, D. (2015). Mindfulness training for adolescents: A neurodevelopmental perspective on investigating modifications in attention and emotion regulation using event‐related brain potentials. Cognitive, Affective, and Behavioral Neuroscience, 15, 696–711. 10.3758/s13415-015-0354-7 PMC4526594 25846954

[desc12646-bib-0060] Sanger, K.L. , & Dorjee, D. (2016). Mindfulness training with adolescents enhances metacognition and the inhibition of irrelevant stimuli: Evidence from event‐related brain potentials. Trends in Neuroscience and Education, 5, 1–11.

[desc12646-bib-0061] Savill, N.J. , & Thierry, G. (2011). Reading for sound with dyslexia: Evidence for early orthographic and late phonological integration deficits. Brain Research, 1385, 192–205. 21316349 10.1016/j.brainres.2011.02.012

[desc12646-bib-0062] Schreiter, S. , Pijnenborg, G.H.M. , & Aan Het Rot, M. (2013). Empathy in adults with clinical or subclinical depressive symptoms. Journal of Affective Disorders, 150, 1–16. 23668900 10.1016/j.jad.2013.03.009

[desc12646-bib-0063] Schultz, W. (2010). Dopamine signals for reward value and risk: Basic and recent data. Behavioral and Brain Functions, 6, 24. 20416052 10.1186/1744-9081-6-24 PMC2876988

[desc12646-bib-0064] Schupp, H.T. , Junghöfer, M. , Weike, A.I. , & Hamm, A.O. (2003). Attention and emotion: An ERP analysis of facilitated emotional stimulus processing. NeuroReport, 14, 1107–1110. 12821791 10.1097/00001756-200306110-00002

[desc12646-bib-0065] Schupp, H.T. , Stockburger, J. , Codispoti, M. , Junghöfer, M. , Weike, A.I. , & Hamm, A.O. (2006). Stimulus novelty and emotion perception: The near absence of habituation in the visual cortex. NeuroReport, 17, 365–369. 16514360 10.1097/01.wnr.0000203355.88061.c6

[desc12646-bib-0066] Shapiro, S.L. , Brown, K.W. , & Biegel, G.M. (2007). Teaching self‐care to caregivers: Effects of mindfulness‐based stress reduction on the mental health of therapists in training. Training and Education in Professional Psychology, 1, 105–115.

[desc12646-bib-0067] Smith, A.R. , Steinberg, L. , & Chein, J. (2013). The role of the anterior insula in adolescent decision making. Developmental Neuroscience, 36, 196–209. 10.1159/000358918 PMC5544351 24853135

[desc12646-bib-0068] Spreng, R.N. , McKinnon, M.C. , Mar, R.A. , & Levine, B. (2009). The Toronto Empathy Questionnaire: Scale development and initial validation of a factor‐analytic solution to multiple empathy measures. Journal of Personality Assessment, 91, 62–71. 19085285 10.1080/00223890802484381 PMC2775495

[desc12646-bib-0069] Sutton, S. , Braren, M. , Zubin, J. , & John, E.R. (1965). Evoked‐potential correlates of stimulus uncertainty. Science, 150, 1187–1188. 5852977 10.1126/science.150.3700.1187

[desc12646-bib-0070] Waxer, M. , & Morton, J.B. (2011). The development of future‐oriented control: An electrophysiological investigation. NeuroImage, 56, 1648–1654. 21316473 10.1016/j.neuroimage.2011.02.001

[desc12646-bib-0071] Way, B.M. , Creswell, J.D. , Eisenberger, N.I. , & Lieberman, M.D. (2010). Dispositional mindfulness and depressive symptomatology: Correlations with limbic and self‐referential neural activity during rest. Emotion, 10, 12–24. 20141298 10.1037/a0018312 PMC2868367

[desc12646-bib-0072] Weir, J.M. , Zakama, A. , & Rao, U. (2012). Developmental risk I: Depression and the developing brain. Child and Adolescent Psychiatric Clinics of North America, 21, 237–259. 22537725 10.1016/j.chc.2012.01.004 PMC3338920

[desc12646-bib-0073] Williams, J.M.G. , Crane, C. , Barnhofer, T. , Brennan, K. , Duggan, D.S. , Fennell, M.J. , … Russell, I.T. (2014). Mindfulness‐based cognitive therapy for preventing relapse in recurrent depression: A randomized dismantling trial. Journal of Consulting and Clinical Psychology, 82, 275–286. 24294837 10.1037/a0035036 PMC3964149

[desc12646-bib-0074] World Health Organization (2015). Data and statistics. Available at: http://www.who.int/maternal_child_adolescent/epidemiology/adolescence/en/

[desc12646-bib-0075] World Health Organization (WHO) (1998). Collaborating Centre for Mental Health, Frederiksborg General Hospital.

[desc12646-bib-0076] Zenner, C. , Herrnleben‐Kurz, S. , & Walach, H. (2014). Mindfulness‐based interventions in schools: A systematic review and meta‐analysis. Frontiers in Psychology, 5, 603. 25071620 10.3389/fpsyg.2014.00603 PMC4075476

